# Modeling of an Optical Sensor Based on Whispering Gallery Modes (WGMs) on the Surface Guiding Layer of Glass Filaments

**DOI:** 10.3390/s8106761

**Published:** 2008-10-28

**Authors:** Wei Tan, Lei Shi, Xianfeng Chen

**Affiliations:** Department of Physics, the State Key Laboratory on Fiber Optic Local Area Communication Networks and Advanced Optical Communication Systems, Shanghai Jiao Tong University, 800, Dong Chuan Road, Shanghai, 200240, China

**Keywords:** Optical sensor, ring resonator, whispering gallery modes

## Abstract

A ring-resonator-based refractive index sensor is proposed in this paper. Glass filaments with surface guiding layers created by ion exchange are crossed with a fiber taper to act as a ring resonator sensor. Theoretical simulation of the sensor response is proposed, and optimization of structural parameters including thickness and refractive index of the surface guiding layer and the diameter of the ring resonator for higher sensitivity is investigated. Results show that a detection limit of a variation of ∼10^-5^RIU can be reached. Due to its simple fabrication and easy manipulation as well as good sensing performance, we believe such a micro-cavity sensor will find potential applications in high sensitivity optical sensing.

## 1. Introduction

Optical ring resonators with Whispering Gallery Modes (WGMs) used as refractive index optical sensors have been intensively investigated in the past few years [1-11]. This kind of sensor detects changes in the surrounding refractive index through the evanescent field traveling outside the ring resonator boundary, providing label-free sensing ability. The light recycling nature and high Q factor resonance of the WGMs significantly enhances the interaction length, thereby greatly reducing both the device size and the amount of sample needed for the detection while achieving high sensitivity.

Micro-ring [1, 2, 3] and micro-disks [4] sensors can be mass fabricated with photolithographic technologies in an array format, but they suffer from limited Q factors due to surface roughness induced scattering loss. Microsphere resonators [5, 6] have much higher Q factors due to their very smooth surfaces. A recently reported silica micro-tube based ring resonator sensor [7-11] demonstrates high sensitivity and high Q factor, as well as good ability to handle aqueous analytes. However, as only the inner surface of such a micro-tube sensor can be used as the sensor surface (the analytes go through the micro-tube), the tube has to be very thin for light to penetrate the inner surface. Typically, it requires a tube thickness of 3 microns to achieve a sensitivity of 2.6 nm/RIU [7]. Significantly higher sensitivity can be obtained with sub-micron wall thicknesses [10]. The fabrication of such a micro-tube sensor involves the tapering of a capillary down to micron or sub-micron thickness, which is rather difficult. Additionally, the micro-tube would be very brittle and difficult to handle in practical sensing applications.

**Figure 1.**
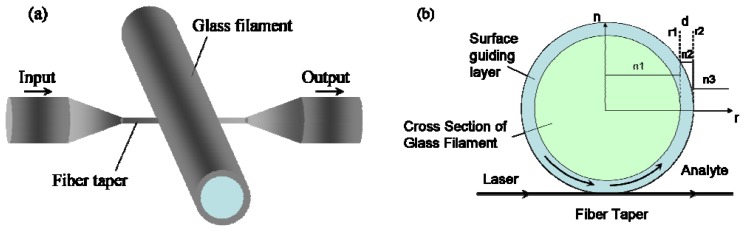
(a) Schematic illustration of the proposed sensor structure. (b) Cross section of the glass filament coupled by a fiber taper, *r*_1_ and *r*_2_ are the inner and outer radius of the surface guiding layer, *n*_1_, *n*_2_ and *n*_3_ are the refractive indices of the core, surface guiding layer and analyte medium respectively.

In this paper we propose a new sensing structure based ona ring resonator formed in glass filaments by ion exchange. The concept of the structure is illustrated in Figure 1. A ring resonator is formed by creating a guiding layer with higher refractive index on the surface of the glass filament through a Ag^+^ ion exchange process [12]. The glass filament is then perpendicularly crossed with a tapered fiber though which light is launched into the WGM of the ring resonator. The sensor detects the refractive index change of the surrounding medium using the evanescent field of WGM near the outer surface. The creation of a guiding layer is to provide confinement of the optical field to the surface of the glass filament, which results in larger evanescent field and enhances the sensitivity of the WGM resonator, as has been investigated extensively by Arnald *et al*. [13, 14]. Glass filaments with tens of microns or even submicron diameter and high surface uniformity can be drawn from bulk glass [15]. The flexible nature of the glass filament makes it easy to manipulate and assembly. The ion exchange process has been used widely to produce waveguides of different dimensions and refractive indices. The fabrication of such a device is relatively simple and it can be readily integrated into an array for enhanced sensing, which may be an advantage over existing refractive index sensors.

## 2. Theory and Simulations

In the following sections of this paper, we simulate the response of the newly proposed sensor to the surrounding refractive index change and optimize the structural parameters for better sensing performance under different conditions, which is of great significance as to provide a theoretical guide for the fabrication of the device and its practical sensing applications.

The radial distribution of the TM WGM can be described by Mie theory [16]:

(1) $$E_{m,l}(r)=\{\begin{array}{ll} AJ_{m}(k_{0}^{\left(l\right)}n_{1}r) & \left({\text{r}}_{1}\geq \text{r}\right)\\ BJ_{m}(k_{0}^{\left(l\right)}n_{2}r)+CH_{m}^{\left(1\right)}(k_{0}^{\left(l\right)}n_{2}r) & \left({\text{r}}_{1}<\text{r}\leq {\text{r}}_{2}\right)\\ DH_{m}^{\left(1\right)}(k_{0}^{\left(l\right)}n_{3}r) & \left(\text{r}\geq {\text{r}}_{2}\right) \end{array}$$

where *J*_m_ and *H*_m_^(1)^ are the *m*th Bessel function and the *m*th Hankel function of the first kind; *m* is the azimuthal mode number and *k*_0_^(^*^l^*^)^ is the amplitude of the wave vector in vacuum for the *l*th-order radial WGM; *n*_1_, *n*_2_ and *n*_3_ are the refractive indices of the glass filament core, the surface guiding layer and the surrounding medium; *r*_1_ and *r*_2_ are the inner and outer radius of the surface guiding layer. By matching the boundary conditions at the inner and outer surface of the guiding layer, the eigen function equation can be obtained, solving which can give us the resonance wavelength. The refractive index change of the surrounding medium *n*_3_ will cause the resonance wavelength of the WGM to shift, which provides the basic sensing mechanism.

The operating wavelength range is set between 1.53∼1.56 μm, for the dual advantage of reusing the standardized telecom equipment and low water absorption [17]. Fluorite glass (SCHOTT LITHOTEC-CAF2, n=1.426) is chosen to be the material of the ring resonator for the convenient phase matching with the silica fiber tapers. The refractive index of the surface guiding layer is assumed to be 1.47 after the ion exchange process.

There are many factors that affect the sensing performance of the proposed sensor, including the selection of WGM mode, the surface guiding layer thickness, the diameter and the refractive index of the ring resonator. The following simulation will be covering these factors.

**Figure 2.**
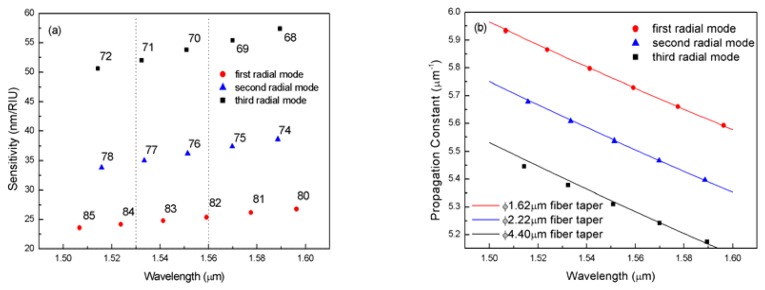
(a) All the modes and their calculated sensitivities within the 1.5∼1.6 μm range. Each mode is identified with its azimuthal mode number *m*. (b) The propagation constants of these modes (dots) and the propagation constant of fiber tapers with different diameters versus wavelength (lines). Parameters *r*_1_=13μm, *r*_2_=15μm, *n*_1_=1.426, *n*_2_=1.47, *n*_3_=1.0.

**Figure 3.**
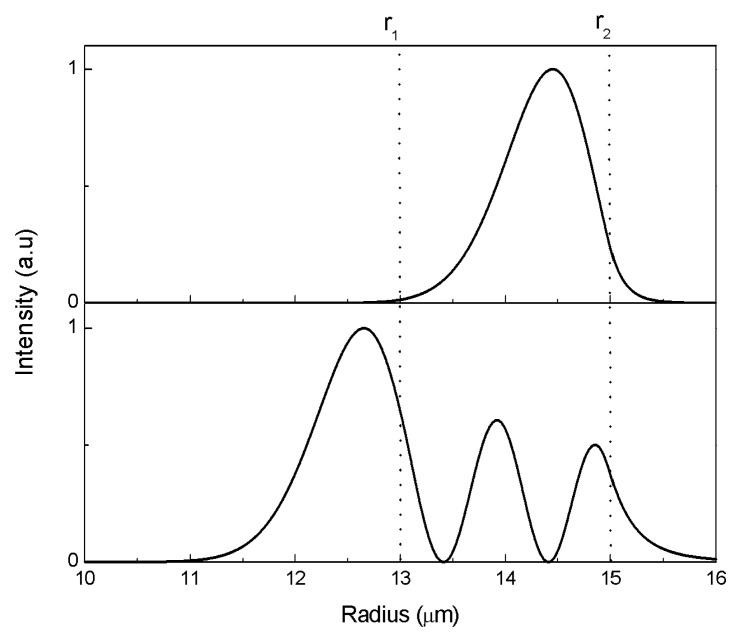
Intensity distribution of the two TM WGMs (*m*=85, *l*=1, top) and (*m*=68, *l*=3, bottom), parameters *r*_1_=13μm,*r*_2_=15μm, *n*_1_=1.426, *n*_2_=1.47, *n*_3_=1.0.

Different WGMs have different sensitivities. The WGMs in Figure 2 include three radial mode families, from bottom to top, the first, second and third radial modes. As we can see from the graph, higher radial order modes have significantly higher sensitivity, while among the same radial modes sensitivity increases with the decrease of azimuthal mode number *m*. Therefore, the WGM with the highest sensitivity within the operating wavelength range is the highest order radial mode with the smallest azimuthal mode number. As shown in Figure 3, higher radial order modes have higher fraction of energy transmitting beyond the outer surface, leading to higher sensitivity. However, higher order radial modes have broader resonant linewidth, which will sacrifice the detection limit. Therefore, in practical applications, both mode sensitivity and mode linewidth should be considered to achieve the best detection limit.

The propagation constant of a WGM is calculated as [18]:

(2) $$\beta =l\int_{0}^{r_{\text{rad}}}\frac{E^{2}}{r}dr/\int_{0}^{r_{\text{rad}}}E^{2}dr$$

where *r*_rad_ is the radiation caustic given by *m*/*n*_3_•*k*_0_. As indicated in Figure 2(b), the propagation constants of one WGM radial mode family can be matched by a fiber taper of a particular diameter. Therefore, the desired WGM can be selected through the phase-matched coupling between the ring resonator and the fiber taper [18-20].

**Figure 4.**
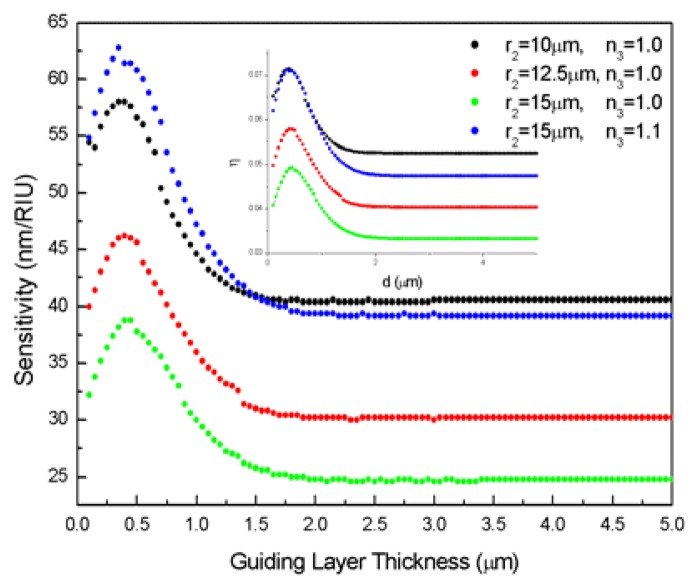
Sensitivity versus surface guiding layer thickness *d*=*r*_2_-*r*_1_ for four different sensor configurations of *r*_2_ and *n*_3_, fixed parameters *n*_1_=1.426, *n*_2_=1.47. Inset: Fractional energy outside the ring resonator.

Then we investigate the effect of different surface guiding layer thickness *d*=*r*_2_-*r*_1_. Figure 4 shows the calculated sensitivity versus *d* under different sensor configurations of *r*_2_ and *n*_3_, while *n*_1_=1.426 and *n*_2_=1.47 remains unchanged. The operating wavelength range is 1.53∼1.56 μm, and the very thin guiding layer can only contain the first radial modes. It can be seen that only *d* values comparable to the wavelength result in an increased sensitivity, and there is an optimal *d* value for a given configuration, where enhancement of sensitivity reaches as much as 50%. This is caused by the radial mode compression into the guiding layer, leaving a larger fraction of mode energy evanescent outside the resonator [14], as shown in the inset of Figure 4. When the inner caustic of the WGM defined by *m*/*n*_2_•*k*_0_ lies between the inner and outer surface of the guiding layer, the inner interface plays a negligible role and the ring resonator operates as a one layer cylinder. The sensitivity drop with *d* value below 0.5μm can be explained by the mode cut-off in the extremely thin surface guiding layer.

**Figure 5.**
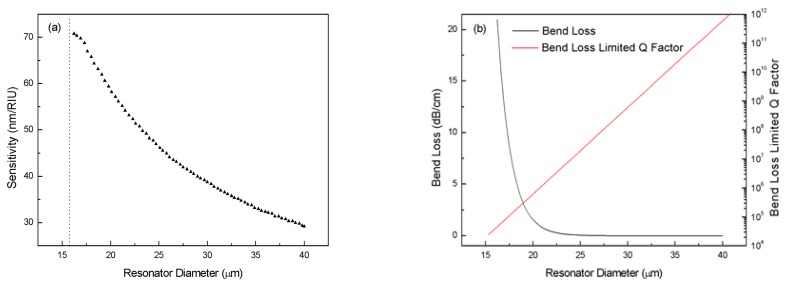
(a) Sensitivity versus resonator diameter. (b) Calculated bend loss and bend loss limited Q factor versus resonator diameter. Parameters *n*_1_=1.426, *n*_2_=1.47, *n*_3_=1.0, *d*=*0*.4 μm.

Next, we calculate the sensitivity of the sensor with different diameters *D*=2*r*_2_. The results in Figure 5(a) indicate that smaller *D* leads to higher sensitivity. However, when the diameter is reduced below a certain value, WGM would cease in the selected operating wavelength range. For *n*_1_=1.426 and *n*_2_=1.47, this diameter is around 16 μm. On the other hand, as the radius gets smaller, bend loss [21] of the resonator increases exponentially and bend loss limited Q factor [22] drops by orders of magnitude, as shown in Figure 5(b). For extremely small diameters below 20 μm, the bend loss limited Q factor goes under 10^6^, which is the typical Q factor of ring-resonator sensors [23], and bend loss becomes the dominating limit for the Q factor. This would greatly compromise the detection limit of the sensor. Therefore, optimization of the sensing ability by reducing resonator diameter is limited by the large bend loss associated with extremely small radius.

**Figure 6.**
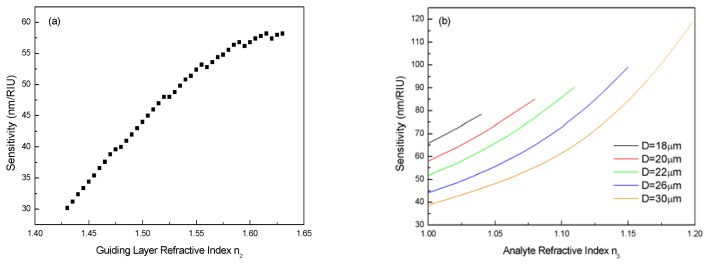
(a) Sensitivity versus guiding layer refractive index, fixed parameters *n*_1_=1.426, *n*_3_=1.0, *d*=0.4μm, *D*=30μm. (b) Sensitivity versus analyte refractive index for five different resonator diameters, fixed parameters *n*_1_=1.426, *n*_2_=1.47, *n*_3_=1.0, *d*=0.4 μm.

The effect of different guiding layer refractive index *n*_3_ is also calculated. From Figure 6(a) one can see that higher *n*_3_ value brings higher sensitivity. The discontinuity in Figure 6(a) is due to the change of the operating WGM for the resonance wavelength to stay in the operating wavelength range.

At last, calculated sensitivity of our device with different diameters versus the analyte refractive index *n*_3_ is plotted in Figure 6(b). It can be seen that higher analyte refractive index possesses significantly higher sensitivity, for higher *n*_3_ would make it easier for the WGM to leak into the surrounding medium. Also, it can be noticed that while demonstrating higher sensitivity, sensor with smaller diameter has smaller measurement range of refractive index. This is also due to mode extinction in the operating wavelength range. Therefore, in practical sensing applications, this trade-off between sensitivity and measurement range should be considered.

The Q factor of the proposed ring-resonator is about 10^5^ [7], which gives a bandwidth of 20 pm. Assuming the resolvable wavelength shift is one-tenth of the resonant bandwidth [24], from the above simulations, the theoretical detection limit of the proposed sensing device is a variation of ∼10^-5^ RIU.

## 3. Conclusions

We have proposed a new structure of refractive index sensor based on ring resonator formed in glass filament by ion exchange and performed simulations of its sensing performance in the 1.53∼1.56 μm operating wavelength range. Results show a detection limit of ∼10^-5^ RIU can be reached and indicates that smaller thickness and larger refractive index of the surface guiding layer and smaller ring resonator diameter lead to higher sensitivity. These simulations will be the theoretical guides for fabrication and application of the device. In addition to its good sensing performance, the simple fabrication of this sensor promises great advantage in practical applications.
